# Immune Recognition of Fungal Polysaccharides

**DOI:** 10.3390/jof3030047

**Published:** 2017-08-28

**Authors:** Brendan D. Snarr, Salman T. Qureshi, Donald C. Sheppard

**Affiliations:** 1 Department of Microbiology and Immunology, McGill University, Montreal, QC H3A 2B4, Canada; brendan.snarr@mail.mcgill.ca; 2 Department of Medicine, Infectious Diseases and Immunity in Global Health Program, Centre for Translational Biology, McGill University Health Centre, Montreal, QC H4A 3J1, Canada; 3 Meakins-Christie Laboratories, Department of Medicine and Division of Experimental Medicine, McGill University, Montréal, QC H4A 3J1, Canada; salman.qureshi@mcgill.ca; 4 Department of Critical Care and Research, Institute of the McGill University Health Centre, Montréal, QC H4A 3J1, Canada

**Keywords:** polysaccharide, immune receptor, fungal cell wall, *Aspergillus fumigatus*, *Candida albicans*, *Cryptococcus neoformans*, *Histoplasma capsulatum*

## Abstract

The incidence of fungal infections has dramatically increased in recent years, in large part due to increased use of immunosuppressive medications, as well as aggressive medical and surgical interventions that compromise natural skin and mucosal barriers. There are relatively few currently licensed antifungal drugs, and rising resistance to these agents has led to interest in the development of novel preventative and therapeutic strategies targeting these devastating infections. One approach to combat fungal infections is to augment the host immune response towards these organisms. The polysaccharide-rich cell wall is the initial point of contact between fungi and the host immune system, and therefore, represents an important target for immunotherapeutic approaches. This review highlights the advances made in our understanding of the mechanisms by which the immune system recognizes and interacts with exopolysaccharides produced by four of the most common fungal pathogens: *Aspergillus fumigatus*, *Candida albicans*, *Cryptococcus neoformans*, and *Histoplasma capsulatum*. Work to date suggests that inner cell wall polysaccharides that play an important structural role are the most conserved across diverse members of the fungal kingdom, and elicit the strongest innate immune responses. The immune system senses these carbohydrates through receptors, such as lectins and complement proteins. In contrast, a greater diversity of polysaccharides is found within the outer cell walls of pathogenic fungi. These glycans play an important role in immune evasion, and can even induce anti-inflammatory host responses. Further study of the complex interactions between the host immune system and the fungal polysaccharides will be necessary to develop more effective therapeutic strategies, as well as to explore the use of immunosuppressive polysaccharides as therapeutic agents to modulate inflammation.

## 1. Introduction

Over the past several decades, there has been a marked increase in the use of immunosuppressive therapy for the treatment of haematologic malignancies, stem cell and solid organ transplantation, and rheumatologic disorders. In parallel, there has also been an increased use of novel surgical techniques, indwelling central venous catheters, and other prosthetic devices in hospitalized patients. These changes in health care, combined with the HIV epidemic, have resulted in a rapid expansion in the number of patients with acquired defects in innate, acquired, and mucosal immunity. This patient population is at increased risk for the acquisition of a wide range of fungal infections, leading to a resurgence of interest in the development of novel antifungal therapeutics.

**Figure 1:**
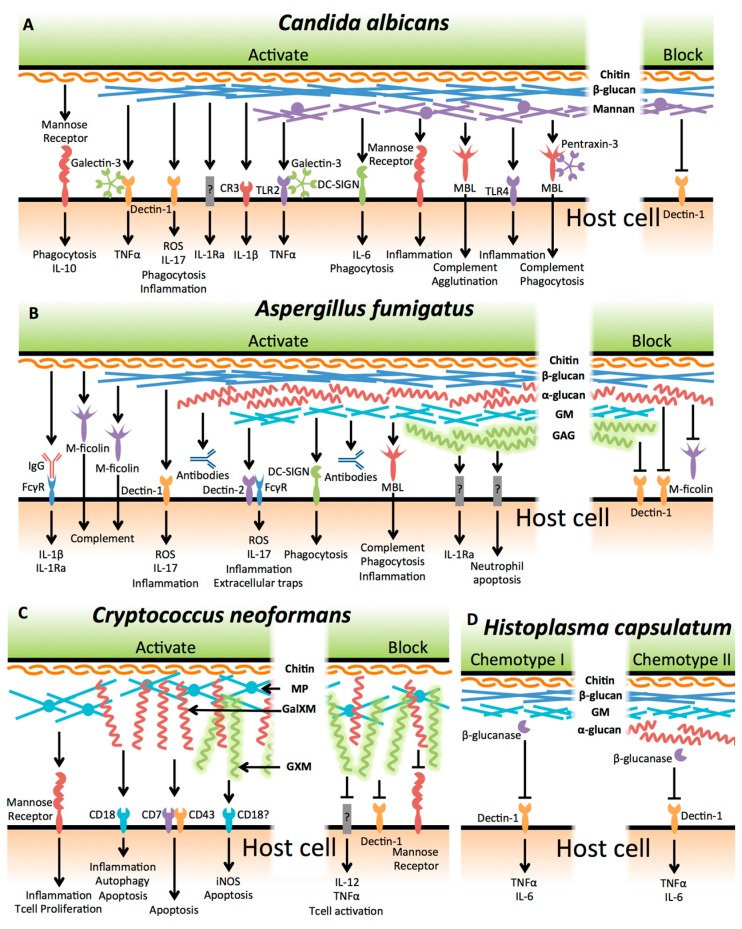
Graphical overview of interactions between fungal polysaccharides and host elements. (**A**) *Candida albicans*; (**B**) *Aspergillus fumigatus*; (**C**) *Cryptococcus neoformans*; and (**D**) *Histoplasma capsulatum*. Abbreviations used: CR3, complement receptor 3; MBL, mannose-binding lectin; GM, galactomannan; GAG, galactosaminogalactan; MP, mannoprotein; GalXM, galactoxylomannan; GXM, glucuronoxylomannan. Green indicates fungal cell, and tan, the host cell.

One approach to combating fungal infections is to augment host recognition and immune response to these organisms. Fungal cell wall glycans and exopolysaccharides play a critical role in these fungal–host interactions. The cell wall is the first point of physical contact between the pathogen and host cells, and fungal polysaccharides have been identified both as ligands for innate immune receptors, and antigens that can stimulate adaptive immune responses. This review will summarize our current understanding of the immune response to fungal exopolysaccharides, and the molecular mechanisms underlying the recognition of these glycans. We have focussed our attention on four of the most common medically relevant fungi: *Candida albicans*, *Aspergillus fumigatus*, *Cryptococcus neoformans*, and *Histoplasma capsulatum* (Figure 1). Studies of immune interactions with the polysaccharides produced by these organisms reveals a common theme. Polysaccharides that are common to multiple fungi, and play a major role in cell wall structure, are associated with the strongest host immune responses through interactions with soluble and cell-associated pattern recognition receptors. However, medically relevant fungi have also developed unique exopolysaccharides that often serve to mask the more conserved glycans from detection by the host, and can even directly downregulate inflammatory responses. Gaps in our current understanding of these host–glycan interactions, and potential areas for future research, will be highlighted.

## 2. *Candida albicans*

*Candida albicans* is a commensal yeast commonly found in the gastrointestinal tract of healthy individuals. However, *C. albicans* is also an opportunistic pathogen that is the most common cause of invasive fungal infection in hospitalized patients [1]. Risk factors for invasive candidiasis include the use of broad-spectrum antibiotics that lead to *C. albicans* overgrowth, surgical and medical interventions that breach normal mucosal barriers to *Candida* invasion, and immunosuppressive illnesses or therapies that impair host immune response [2]. Additionally, *Candida* can form biofilms on biomedical devices, including urinary and vascular catheters [3]. During infection, *C. albicans* can switch morphologies between yeast cells, pseudohyphae and hyphae [4]. This ability to undergo morphogenesis is a critical virulence factor of *C. albicans*, and allows it to exploit a variety of environmental niches within the host [4]. The interactions of the host immune system with *C. albicans* glycans are the best studied among the medically relevant fungi, and have greatly advanced our understanding of innate immune recognition of these organisms.

### 2.1. Beta-Glucan

β-glucans are key structural polysaccharides found largely within the *C. albicans* inner cell wall. β-(1,3)-linked glucans are the most abundant of these glucans, with shorter chains of β-(1,6)-glucan that serve to cross-link the inner and outer cell wall [5]. β-(1,3)-glucans are pathogen associated molecular pattern (PAMP) ligands that are recognized by the pattern recognition receptor (PRR) dectin-1 (also known as CLEC7A) [6]. This interaction is the best studied of all fungal–innate immune interactions, and is common to most pathogenic fungi.

Dectin-1 is a transmembrane C-type lectin that is primarily expressed on the surface of immune cells, such as dendritic cells, alveolar macrophages, and neutrophils. The extracellular portion of dectin-1 consists of a carbohydrate recognition domain (CRD) atop a stalk region, while the intracellular portion contains an immunoreceptor tyrosine-based activation motif (ITAM) involved in signalling [7]. The CRD of dectin-1 recognizes β-(1,3)-glucan fragments that are a minimum of seven glucose residues long [8]. Dectin-1 activation is proportional to β-glucan polymer length, and it has been suggested that dectin-1 activation requires receptor clustering as part of the phagocytic synapse [8,9]. Dectin-1 signalling induces phosphorylation of Syk and IκB, and translocation of NF-κB to the nucleus [10,11], as well as Syk-independent signalling through Raf1 activation [12]. Dectin-1 activation controls a wide range of immune responses, including regulation of leukocyte phagocytosis, recruitment of Toll-like receptor (TLR) 9 to phagolysosomes, phagolysosome maturation, activation of autophagy, production of reactive oxygen species (ROS), activation of the inflammasome, and induction of pro- and anti-inflammatory cytokine secretion ([13,14], and reviewed in [15]).

Dectin-1 signalling in response to β-glucan can be modulated by interaction with other host proteins. The soluble galactose-specific lectin galectin-3 (previously known as Mac-2, εBP, or CBP30/35) physically interacts with dectin-1, likely through binding of glycosylated domains of the receptor to form multivalent oligomers that enhance clustering and activation of dectin-1 [16]. Galectin-3 exists in both a cytoplasmic and secreted form, and it remains to be determined if interaction of this lectin with dectin-1 occurs in the intracellular or extracellular space. Galectin-3 knockdown and overexpression studies in macrophages suggest that galectin-3 interaction with dectin-1 augments TNF production in response *to C. albicans* β-glucan [17]. However, the significance of this specific interaction is unclear, since, as detailed below, galectin-3 also interacts with *C. albicans* mannans [18], and there are conflicting reports as to the role of galectin-3 in host defence in vivo. A recent study reported that galectin-3 deficiency in mice increased resistance to *C. albicans* infection, and that intracellular galectin-3 suppressed Syk signalling within neutrophils to decrease ROS production [19]. These results contrast with an earlier report in which galectin-3 deficiency was associated with increased susceptibility of mice to *C. albicans* infection [20]. The molecular mechanisms underlying these conflicting studies are unknown, but may reflect different roles of intracellular and extracellular galectin-3 in the modulation of immune responses.

Other host receptors have been found to participate in the recognition and response to β-glucan. Complement receptor 3 (CR3, also known as Mac-1 and integrin α_M_β_2_) enhances fungal responses through recognition of β-(1,6)-glucan [21]. In mouse bone marrow-derived neutrophils, CR3 expression is upregulated following dectin-1 recognition of β-glucan particles, resulting in phagocytosis of the particles and ROS production [22]. While CR3 is not required for phagocytosis of β-glucan particles by mouse peritoneal macrophages [22], IL-1β release by mouse bone marrow-derived macrophages (BMDMs) and bone marrow derived dendritic cells (BMDCs), in response to purified β-glucan and heat-killed *C. albicans*, is dependent on CR3 [23]. Consistent with these findings, CR3-deficent mice exhibit higher mortality and fungal burden compared to wild-type animals, when challenged with *C. albicans*, highlighting the importance of this receptor in host defence [22].

It is likely that other host receptors participate in the recognition of β-glucan. For example, β-glucan on the surface of *C. albicans* hyphae was reported to induce the production of IL-1 receptor antagonist (IL-1Ra) by peripheral blood mononuclear cells (PBMCs) via a dectin-1 and CR3-independent pathway [24]. While the mechanism of IL-1Ra induction is currently not well understood, inhibition of Akt and PI3K significantly reduced IL-1Ra production [24]. The identification and characterization of novel β-glucan recognition receptors is an area of great interest for future studies.

There is also evidence to suggest that some *C. albicans* strains are recognized by the host by dectin-1/β-glucan independent mechanisms. Support for this hypothesis comes from a screen of 14 *C. albicans* strains from a range of clades, which were analyzed for their ability to stimulate cytokine release by human peripheral blood mononuclear cells (PBMCs) [25]. One strain induced PBMC IL-1β production that was inhibited by blocking of the mannose receptor (see below), but was unaffected by dectin-1 blockade [25]. Interestingly, this strain was hypovirulent in an intravenous mouse infection model [26], and failed to stimulate KC and MIP-2 production by M-1 murine renal epithelial cells, or mediate cellular damage in vitro [27]. Taken together, these findings suggest that some strains of *C. albicans* have developed adaptations to evade dectin-1 detection, but these changes are associated with a loss of virulence.

In addition to natural recognition of β-glucan by the innate immune system, efforts have been made to use these glycans to augment adaptive immune responses against fungi. A β-glucan vaccine was generated by conjugating laminarin, composed of a β-(1,3)-glucan backbone with β-(1,6)-glucan branches isolated from the alga *Laminaria digitata*, to the diptheria toxoid CRM197 [28]. Immunization with this antigen increased mouse survival from 10 to 70%, as compared to the adjuvant alone, in a model of systemic candidiasis [28]. Protection was antibody-mediated, as treating naïve mice with either serum from vaccinated mice, or a monoclonal IgG antibody raised against the β-glucan antigen, was effective at limiting fungal burden and improving survival. These antibodies reduced *C. albicans* adherence to human epithelial cells, as well as inhibited fungal growth in vitro [28,29]. The β-glucan vaccine provided only modest protection in a model of vaginal candidiasis, likely due to poor penetration of β-glucan-specific IgG antibodies to the vaginal mucosa [30]. However, vaccinated mice exhibited increase resistance to intravenous challenge with *Aspergillus fumigatus* [28] and intravenous challenge with *Cryptococcus neoformans* [31], highlighting the potential of β-glucan immunization to protect against a broad range of fungal pathogens.

### 2.2. Mannan

The outer layer of the *C. albicans* cell wall is composed of an array of heavily mannosylated proteins that are glycosylphosphatidylinositol (GPI)-modified and cross-linked to β-(1,6)-glucans [32,33]. Gas chromatography and nuclear magnetic resonance studies have suggested that *N*-linked mannans are large branched structures that consist primarily of an α-(1,6)-mannose backbone with α-(1,2)-oligomannose sidechains that are commonly capped with β-(1,2)-linked mono-, di-, tri, or tetramannans [34]. Genetic studies have suggested that phospholipomannans comprised of β-(1,2)-oligomannans can also be attached to the *N-*linked mannans via β-(1,2)-mannosyltransferases [35]. In contrast, *O*-linked mannans of *C. albicans* are primarily linear α-(1,2)-oligomannans [34]. The relative abundance and composition of the mannans differs between *C. albicans* yeast and hyphae, with reduced phosphodiesterification of the β-(1,2)-oligomannans and decreased branching of the α-(1,6) backbone in hyphae [34].

Multiple studies have demonstrated that the outer mannan layer plays an important role in concealing β-glucans from host immune detection. Treatment of mouse BMDCs with heat-killed yeast producing *N*-linked mannans deficient in β-(1,2)-mannan, resulted in the release of higher levels of pro-inflammatory cytokines, such as IL-6, IL-12p40, IL-23, and TNFα, as compared with wild type *C. albicans* [36]. Similarly, live yeast cells deficient in mannan branching induced greater levels of IL-1β, IL-10, and TNFα secretion by human PBMCs, largely due to enhanced β-glucan exposure [37]. Deletion of α-(1,6)-mannosyltransferase in *C. albicans* produced a strain with severely-truncated *N*-linked mannans and increased β-glucan exposure, which resulted in higher IL-6 and TNFα production by mouse peritoneal macrophages [38]. Similarly, a *C. albicans* strain deficient in *O*-linked mannans exhibited increased β-glucan exposure, and was unable to prevent phagolysosome maturation in RAW and J774 macrophage cell lines, as well as mouse peritoneal macrophages, resulting in a reduced ability of *C. albicans* to lyse and escape from these cells [39]. Finally, chemical removal of the mannan layer of the cell wall resulted in enhanced activation of the alternative pathway of the complement cascade, as determined by the ability of human neutrophils to phagocytize yeast cells in the presence of human serum [40].

Although mannans play an important role in immune evasion, a number of host receptors can directly recognize mannans and augment host defences. The C-type lectin, dendritic cell-specific intercellular adhesion molecule 3-grabbing nonintegrin (DC-SIGN, also known as CD209), was found to bind to *N*-linked mannans [41]. DC-SIGN is comprised of an extracellular stalk consisting of multiple CRDs, and an intracellular domain containing motifs that participate in internalization [42]. The CRD of DC-SIGN shows specificity for mannose-containing oligosaccharides [43]. These multiple CRDs are thought to aid in receptor multimerization and clustering [44]. Consistent with the internalization motifs on the cytosolic domain, DC-SIGN is thought to function largely as an endocytic receptor, and is primarily expressed on professional antigen-presenting cells, such as macrophages and dendritic cells [42]. Phagocytosis of *C. albicans* yeast cells by human monocyte-derived dendritic cells, and release of IL-6, is dependent on DC-SIGN and *N*-linked mannans [41,45].

*N*-linked mannans are also recognized by a number of other receptors. The mannose receptor (MR, also known as CD206), a transmembrane lectin found predominantly in macrophages [46], can recognize terminal mannose structures [47]. While the MR is primarily expressed on the cell surface, a soluble form can also be released through proteolytic cleavage [47]. TLR4, on both human mononuclear cells and murine macrophages, recognizes *O*-linked mannans on the surface of *C. albicans* yeast cells [46]. Optimal release of TNFα, IL-6, IL-10, and interferon (IFN)γ by these cells required recognition of both *N*- and *O*-linked mannans by MR and TLR4, respectively [46]. Human mononuclear cells incubated with *C. albicans* deficient in either *N*- or *O*-linked mannans produced lower levels of cytokines, which could be further reduced with blocking antibodies targeting the receptor for the other mannan structure [46]. Finally, dectin-2 (also known as CLEC6A or CLECSF10), a C-type lectin commonly expressed by tissue macrophages, dendritic cells, and PBMCs, has also been implicated in the recognition of *C. albicans* mannans [48]. The CRD of dectin-2 is specific for α-(1,2)-mannan structures, which are significantly masked by the β-linked mannan caps [49]. Mouse peritoneal macrophages exhibited increased IL-6 and KC release in vitro, in response to *C. albicans* lacking β-mannans, in a dectin-2-dependent manner [49]. Importantly, dectin-2 lacks a cytoplasmic signalling motif, and therefore, must associate with other receptors to transduce signals. Dectin-2 forms heterodimers with dectin-3 (also known as CLEC4D or CLECSF8), another C-type lectin that recognizes α-mannans, to activate intracellular signalling cascades [50]. This heterodimeric complex is thought to recruit the Fcγ receptor (FcγR) to further activate signalling cascades [50,51,52]. RAW264.7 macrophages, stably expressing both dectin-2 and dectin-3, produced greater amounts of TNF-α in response to purified α-mannans, than did cells expressing only one of the receptors [50]. Dectin-2-deficient mice are hypersusceptible to intravenous challenge with both *C. albicans* and *Candida glabrata*, exhibiting decreased survival, greater kidney fungal burden, and reduced production of T helper (T_H_) 1- and T_H_17-derived cytokines by splenocytes, as compared to the wild type control mice [49,53]. Dectin-3-deficient mice were also hypersusceptible to *C. albicans* intravenous infection, with higher mortality and kidney fungal burden as compared with wild type mice [50], illustrating the importance of the dectin-2/dectin-3 heterodimer in recognizing α-mannans and mounting a successful anti-fungal immune response.

Soluble receptors can also interact with *Candida* mannans. The β-(1,2)-mannan sidechains of the *N*-linked mannans on *C. albicans* yeast cells were specifically recognized by galectin-3 [18]. While phagocytosis by J774 macrophages was not found to be dependent on galectin-3, the interaction between galectin-3 and β-(1,2)-mannan was required for optimal TNFα release by both J774 cells and mouse peritoneal macrophages [18]. This signalling occurred via TLR2 activation in macrophages, and may provide a mechanism for the host to discriminate between pathogenic *C. albicans* and the commensal *Saccharomyces cerevisiae*, which lacks these β-(1,2)-mannan sidechains [18]. Intriguingly, it has been reported that binding of human recombinant galectin-3 to β-(1,2)-mannan can directly kill *C. albicans* yeast cells in the absence of any other immune effectors [54], although the mechanisms underlying this observation remain unknown.

Mannose-binding lectin (MBL, also known as MBL2) is likely another important host receptor for *C. albicans* mannans. MBL is a soluble circulating collectin-class lectin of hepatic origin that forms helical quaternary structures that increase its avidity for carbohydrates [55]. Upon binding to carbohydrates, MBL induces complement activation via complex formation with MBL-associated serine proteases (MASPs) [56]. While the precise carbohydrate ligand remains unclear, human recombinant MBL bound strongly to budding and young yeast cells, as well as hyphae [57]. This binding was temperature-specific, as MBL bound strongly to mature yeast grown at 37 °C, but not to yeast grown at 23 °C [57]. Mannans purified from cultures grown at 23 °C were recognized by MBL, suggesting that failure to detect MBL ligand at this temperature is due to masking by another polymer [57]. As with galectin-3, MBL binds poorly to *S. cerevisiae*, suggesting a role in discriminating between commensal and pathogenic fungi [57]. Human MBL initiates the agglutination of *C. albicans* hyphae [58], and can recruit additional host factors to aid in immune recognition of these fungal structures. Binding of MBL to the cell wall resulted in enhanced deposition of complement proteins C4 and C3b from normal human serum, and augmented phagocytosis of yeast by human blood polymorphonuclear cells (PMN) [59], but not human monocyte-derived DCs. These actions are likely due to heterocomplexes formed between MBL and other serum proteins, as complexes of MBL and either pentraxin-3 or serum amyloid P, result in C4 and C3b-mediated human PMN phagocytosis of *C. albicans* yeast [60]. As with galectin-3, MBL binding to *C. albicans* also directly inhibits growth of the fungus, suggesting that they may bind similar glycans on the fungal cell wall [58]. The MBL-pentraxin-3 heterocomplex activates complement-mediated killing through deposition of complement protein C1q [60]. Taken together, these findings suggest that MBL mediates a complex anti-*C. albicans* response through both complement-mediated killing and opsonisation. Consistent with these observations, prophylactic treatment of mice with MBL improves survival following intravenous *C. albicans* challenge [57]. Furthermore, genome-wide association studies have identified polymorphisms in the human *MBL* gene, with heightened susceptibility to vulvovaginal candidiasis and increased rates of recurrence of this condition [61], suggesting a role for *MBL* in mucosal immunity against *C. albicans*.

Several studies have evaluated the virulence of mannan-deficient strains of *C. albicans* [38,46,62]. Strains deficient in *O*-linked mannan had slower growth rates and greater antifungal susceptibility, suggesting that *O*-linked mannosylation may also be required for normal mannoprotein function. Consistent with these observations, *O*-linked mannan-deficient strains exhibited attenuated virulence in a mouse model of systemic infection [62]. Mice infected with *N*-linked mannan-deficient *C. albicans* exhibited higher survival and lower kidney fungal burden, as well as reduced levels of kidney IL-6 and TNFα, as compared to those infected with wild-type *C. albicans* [46]. Similarly, despite no observed defect in growth rate, an α-(1,6)-mannosyltransferase-deficient mutant that produces a severely-truncated *N*-linked mannan backbone, also exhibited attenuated virulence in a mouse model of systemic candidiasis, in association with increased T_H_1 and T_H_17 responses and increased kidney levels of IFN-γ, IL-6, and IL-17 [38]. Thus, despite the presence of a range of host receptors that can mediate recognition of *Candida* mannans, these animal studies suggest that the virulence promoting characteristics of these polysaccharides predominate during invasive infection.

### 2.3. Chitin

Chitin is a ubiquitous exopolysaccharide composed of β-(1,4)-*N*-acetylglucosamine that is produced by a wide array of arthropods, parasites, and fungi. Chitin is found within the innermost layer of the *C. albicans* cell wall [33], where it plays an important role in maintaining cell rigidity and resistance to physical stress. While it is one of the least well-studied cell wall components of *C. albicans*, several recent studies using purified *C. albicans* chitin have begun to shed light onto the immunomodulatory effects of this glycan. Pure chitin is a strong activator of the complement cascade, and can induce cleavage of complement protein C3 via the alternative complement pathway to produce C3a, a potent anaphylatoxin [63]. Human PBMCs pre-treated with chitin produced lower levels of the pro-inflammatory cytokines TNFα, IL-6, and IL-1β in response to *C. albicans* yeast exposure [64]. Treatment of *C. albicans* with sub-therapeutic concentrations of caspofungin, which increased the surface exposure of chitin, also resulted in a reduced pro-inflammatory cytokine response [64]. While the authors reported no difference in β-glucan content in caspofungin-treated and untreated *C. albicans* [64], it is difficult to exclude the possibility that inhibitory effects of this agent on β-glucan synthesis may have also have contributed to these observations [65]. Treatment of mice with intranasal chitin induced release of IL-25 and IL-33 by airway epithelial cells, resulting in type-2 innate lymphoid cell production of IL-5, and pulmonary recruitment of eosinophils and M2 macrophages [66]. Intraperitoneal administration of chitin induced eosinophilia and suppressed the TNFα response to LPS challenge in mice [67]. Similarly, purified *C. albicans* chitin directly enhanced the release of the anti-inflammatory cytokine IL-10 by human PBMCs [67]. IL-10 production in these studies was dependent on the MR, and involved TLR9 and NOD2 activation [67]. Chitin-mediated anti-inflammatory responses have been postulated as a mechanism of resolving inflammation when non-viable chitin “ghosts” remain following successful killing of the *C. albicans* yeast cells [67].

### 2.4. Candida albicans Biofilms

During infection, *C. albicans* commonly grows in biofilms formed on prosthetic devices or mucosal surfaces [68,69]. While the majority of studies examining the host response to *C. albicans* have been performed using planktonic cells, there have been recent efforts to examine the immune response to biofilm-grown organisms (reviewed in [70]). Cytokine production by PBMCs has been reported to differ between biofilm and planktonic cells with high levels of IL-1β, IL-10, and MCP-1, and lower IL-6 and MIP1β production, in response to biofilm-grown organisms [71]. Impaired phagocytosis and killing of biofilm-associated organisms by PBMCs [71], monocytes [72], and neutrophils [72,73,74] have all been reported. Impaired activation of neutrophils has been linked to β-glucans [74], as well as the GPI-anchored cell wall protein Hyr1 [75]. *Candida* biofilms formed on mucosal surfaces are characterized by the infiltration of abundant neutrophils [76,77,78,79,80], a process that has been linked to the production of chemotactic factors, such as alarmins, by epithelial cells in response to fungal colonization [77,81,82,83].

### 2.5. Non-albicans Candida Species

While *C. albicans* are the most common species isolated from *Candida* infections, rates of infections with other *Candida* species are increasing [84]. Among these species, *C. glabrata* and *C. parapsilosis* are the most frequently implicated in nosocomial infections [84]. Differences in host–pathogen interactions between these strains have been reported in a number of studies.

*C. glabrata* is more closely genetically related to the non-pathogenic yeast *Saccharomyces cerevisiae*, and produces surface mannans more closely related to this organism [85,86]. In contrast to *C. albicans*, disruption of mannosyltransferases that mediate synthesis of *N*-linked mannans enhanced the virulence of these strains [87], suggesting that recognition of these glycans by host PRRs is important in innate host defence. J774 macrophages phagocytosed *C. glabrata* more avidly than *C. albicans* [88], and *C. glabrata* survival within the phagosomes of macrophages has been linked to decreased chitin exposure [89]. Whether this chitin masking is a consequence of cell wall mannans remains to be determined.

Although less is known about the cell wall composition of *C. parapsilosis*, lectin staining suggests differences in chitin exposure as compared with *C. albicans* [90]. Deletion of the α1,6-mannosyltransferase, responsible for initiating *N*-linked mannan production, resulted in a strain that induced higher levels of pro-inflammatory cytokine production by PBMCs [91]. Increased cytokine production was associated with increased exposure of β-glucans, and was reduced by laminarin-mediated blocking of dectin-1 or with antibodies to TLR4 [91]. As with *C. albicans*, loss of *N*-linked mannans was associated with attenuated virulence [91]. In contrast, β-elimination trimming of *O*-linked mannans increased production of IL-10 by PBMCs stimulated with live wild type cells, and reduced pro-inflammatory cytokine induction by *N*-linked mannan-deficient organisms [91]. Collectively, these observations suggest a pro-inflammatory role for *O*-linked mannans of *C. parapsilosis*, although further studies are required to validate these observations in vivo.

## 3. *Aspergillus fumigatus*

*Aspergillus fumigatus* is an ubiquitous mould that produces abundant airborne conidia. Every day, humans inhale up to one hundred of these conidia, which are largely eliminated by the airway cell mucociliary action or killed by pulmonary macrophages, before they undergo germination [92]. Dormant conidia are coated in a layer of hydrophobic rodlet proteins that are largely immunoinert and conceal cell wall polysaccharides from immune detection [93]. If conidia evade these initial immune defences, they begin to swell and shed this layer of hydrophobins, exposing deeper cell components. Swollen conidia then undergo germination to produce filamentous hyphae, which can invade host tissues and blood vessels [94]. The cell wall composition of each of these fungal growth stages differs [93], and as a result, the host exhibits stage-specific immune responses to *A. fumigatus*. Despite current antifungal therapies, the mortality of invasive aspergillosis remains between 50% and 90%, highlighting the need for new treatment options for this infection [95]. Strategies targeting cell wall polysaccharide synthesis, and the immune response to these glycans, are two promising therapeutic approaches.

### 3.1. Beta-Glucan

Mutants devoid of β-glucan are viable, but produce leaky, fragile cell walls, and are markedly impaired in growth and development [96]. In resting conidia, β-glucan is concealed by a layer of hydrophobic proteins, termed rodlets [93]. During germination, conidia shed this rodlet layer to display high levels of surface exposed β-glucan [97], leading to an increased production of dectin-1-dependent CXCL1, CXCL2, and TNFα by BMDMs [98]. Alveolar macrophages isolated from dectin-1-deficient mice were impaired in their ability to produce proinflammatory cytokines, such as IL-1α, IL-1β, TNFα, MIP-1α, MIP-1β, and KC in response to live *A. fumigatus* conidia after 24 h of growth [99], a finding that has been validated in vivo in an *Aspergillus* keratitis model [10]. Similarly, human monocyte-derived dendritic cells incubated with young hyphae exhibited significantly reduced expression of IL-12 and TNFα when treated with either dectin-1 blocking antibodies or transfected with dectin-1 silencing RNA [100]. Additionally, thioglycolate-elicited neutrophils from dectin-1-deficient mice produced lower levels of ROS when challenged with swollen *A. fumigatus* conidia, and exhibited impaired killing of the fungus in vitro [99]. As hyphae mature, β-glucan is again masked by the production of the exopolysaccharide galactosaminogalactan (discussed further below) [101]. 

Dectin-1 is required for normal production of IL-23 by dendritic cells in response to *Aspergillus* [102]. Production of IL-23 plays an important role in defence against fungal infection through stimulating neutrophil IL-17 production. Mice deficient in dectin-1 produced lower levels of IL-17A, exhibited reduced neutrophil recruitment to the site of infection, and had increased mortality following pulmonary challenge with *A. fumigatus* [99]. Dectin-1-dependent IL-23 secretion was also required for optimal IL-22 responses in a mouse model of pulmonary aspergillosis [99]. IL-22 induction was necessary for optimal IL-1α, IL-12 (both p40 and p70), CCL3, CCL4, and TNFα release, leading to control of fungal infection [103]. These findings have been corroborated with a model of fungal keratitis, where dectin-1 was required for optimum IL-1β and KC production, and control of fungal growth [10]. These dectin-1-mediated responses are most important against germinating conidia and young hyphae, as β-glucans are cloaked by the exopolysaccharide galactosaminogalactan produced by growing hyphae (discussed further below) [97,101]. 

Dectin-1 also plays a role in facilitating the adaptive immune response to *A. fumigatus*. Dectin-1-deficiency resulted in alterations in *A. fumigatus*-specific T cell maturation following adoptive transfer and pulmonary challenge with *A. fumigatus* [104]. Analysis of bronchoalveolar lavage (BAL) fluid from these mice revealed a greater abundance of IL-17-producing T cells in wild-type mice than was found in dectin-1 deficient animals in which IFN-γ-positive T cells were most abundant [104]. In a mouse model of *A. fumigatus*-induced allergy, production of IL-17, IL-4 and IL-13 by T cells was dectin-1-dependent, leading to increased airway resistance and allergic pathology [105]. These dectin-1-dependent effects were mediated by IL-22 production, illustrating that while this cytokine is beneficial in the context of acute *A. fumigatus* infection, it can be detrimental in allergic disease.

As with *C. albicans*, other host molecules are thought to participate in the detection and response to β-glucans during *A. fumigatus* infection. M-ficolin, also known as ficolin-1, is a member of the ficolin family of opsonins that mediate recognition of pathogens *Escherichia coli* and *Staphylococcus aureus*, and activation of the complement pathway [106]. Recombinant human M-ficolin binds to conidia and young hyphae of *A. fumigatus*, to β-(1,3)-glucan-containing *A. fumigatus* alkali-insoluble hyphal cell wall fraction (AIF) [107], and to purified β-(1,3)-glucan [108]. M-ficolin binding to AIF and purified β-(1,3)-glucan activates the lectin-dependent complement pathway in vitro [108], and enhances IL-8 secretion by A594 airway epithelial cells, when incubated in vitro with AIF [108]. A synergistic interaction between M-ficolin and the soluble pattern recognition receptor pentraxin-3 has also been observed in vitro, resulting in greater M-ficolin binding to the β-glucan of *A. fumigatus*, and C4 deposition and activation of the complement cascade [109]. While M-ficolin has been detected in granulocytes and monocytes at the periphery of pulmonary aspergillomas in humans [108], the role of M-ficolin during experimental *A. fumigatus* infection has not been studied.

### 3.2. Galactomannan

*A. fumigatus* galactomannan (GM), is composed of an α-(1,2)(1,6)-mannopyranose backbone with short branches of β-(1,5)-oligogalactofuranose connected by β-(1,3) and β-(1,6) linkages [110]. GM is found in the hyphal cell wall, conjugated to both proteins [111] and glucans [112], as well as in a soluble form that is shed into the environment. Additionally, a second species of GM, produced by the action of a unique set of mannosyltransferases, is present exclusively in the conidia, where it appears to be involved in conidial separation during sporulation [112]. A mutant lacking these mannosyltransferases produced conidia with altered cell wall organization and reduced viability [112]. The solubility of GM, as well as its relative specificity for *Aspergillus* species, makes it a useful diagnostic marker of *Aspergillus* infection [113,114].

The mannan and galactofuranose components of GM are differentially recognized by the host. The mannan core closely resembles cell wall mannans of other fungi, and as a consequence, interacts with many of the host mannose receptors described above. The best described receptor for the mannan core is DC-SIGN [115]. Antibodies to DC-SIGN dramatically reduce binding and phagocytosis of conidia by human monocyte-derived dendritic cells [116]. While the ligand interacting with DC-SIGN was not defined in this study, purified *A. fumigatus* GM was found to block binding of conidia to dendritic cells [116,117]. Surprisingly, DC-SIGN played no role in the cytokine response of human monocyte-derived dendritic cells to *A. fumigatus* young hyphae, as DC-SIGN knockdown experiments revealed no change in *TNFA* or *IL12* gene expression, as compared with vector controls [100]. There are several possible interpretations for these results. It is possible that *Aspergillus* mannan is not a potent inducer of cytokine responses, or that other mannan receptors can compensate for the loss of DC-SIGN. An alternate, intriguing hypothesis is that DC-SIGN is more specific for the recognition of the unique species of soluble GM found in conidia, which were not tested in this study [112].

Dectin-2 also plays an important role in the detection of the α-mannan backbone of GM. Binding of swollen conidia and hyphae by THP-1 macrophages is dectin-2-dependent, leading to Syk-dependent signalling and NF-κB-specific activation [118]. These NF-κB-dependent responses include release of IL-1β, IL-10, IL-23, and TNFα, as well as the generation of ROS [118]. These responses were not observed in response to resting conidia, likely due to the rodlet layer of the conidia masking cell wall GM [98]. Consistent with this hypothesis, rodlet-deficient mutants induced higher levels of CXCL2 and TNFα production by BMDMs in a dectin-2-dependent manner [98]. Recognition of *A. fumigatus* hyphae by human plasmacytoid dendritic cells was also found to be dectin-2 dependent, leading to release of TNFα and IFN-α [119], and production of extracellular traps by these cells [119]. In mouse bone marrow neutrophils, dectin-2 surface expression was induced in response to IL-6 and IL-23, where it augmented IL-17 release, leading to increased killing of *A. fumigatus* hyphae in vitro [120]. Finally, human data support a role for dectin-2 in the pathogenesis of invasive aspergillosis, as increased expression of dectin-2, largely restricted to macrophages, was observed during pulmonary infection [121].

Although MBL binding to GM has not been specifically demonstrated, purified MBL binds to the surface of *A. fumigatus* resting conidia [122], an interaction that could be inhibited with mannose, N-acetylglucosamine, and EDTA [123]. Human corneal epithelial cells up-regulate and secrete MBL in response to *A. fumigatus* antigens [124], and MBL enhances phagocytosis of conidia, and killing of *A. fumigatus* hyphae by human PMNs in the presence of serum [125]. As with *C. albicans*, interactions between MBL and *A. fumigatus* activate the complement cascade, however, there are conflicting reports in the literature as to whether this occurs via C4 deposition [125], or the C2 bypass mechanism [122].

Mouse models have suggested site and condition-specific roles for MBL in the pathogenesis of *Aspergillus* disease. In a model of invasive pulmonary aspergillosis, a single dose of 0.05 mg/kg of recombinant human MBL increased mouse survival from 0 to 80% [125]. MBL-mediated protection was associated with increased splenocyte production of TNFα and IL-1β, and decreased IL-10 production [125]. In contrast, during intravenous *A. fumigatus* infection, MBL-deficient mice were more resistant to fungal challenge [126]. In a model of *A. fumigatus*-induced asthma, MBL-deficient mice exhibited significantly lower production of type-2 cytokines and reduced airway hyperresponsiveness at 4 days post challenge, suggesting that MBL contributes to the allergic response towards *Aspergillus* [127]. However, by 28 days post challenge, minimal differences were observed between wild type and MBL-deficient mice [127], suggesting that MBL is not involved in the airway remodeling seen in chronic fungal asthma. In humans, polymorphisms resulting in reduced MBL expression have been associated with chronic necrotizing pulmonary aspergillosis [128]. Taken together, these findings suggest that MBL-mediated recognition of *A. fumigatus* mannans is likely important during early pulmonary host–fungal interactions.

While the mannan core of GM is not recognized by sera from aspergillosis patients [110], the oligogalactofuranose side chains of GM are antigenic in experimental animals [129]. An anti-galactofuranose monoclonal antibody forms the basis for the non-culture based *Aspergillus* antigen EIA, which has revolutionized the early diagnosis of invasive aspergillosis in immunocompromised patients [130,131]. No host receptors specific for *A. fumigatus* oligogalactofuranose have been described to date.

### 3.3. Alpha-Glucan

The α-(1,3)-glucan of *A. fumigatus* is found within the outer cell wall during growth in vitro, where it is involved in cell wall stability and agglutination of germinating conidia and hyphae [132,133,134]. However, the role of α-glucan in mediating aggregation may vary by morphology and environment, as electron microscopy studies of hyphae during pulmonary infection localized α-glucan largely within the inner cell wall of hyphae [135].

No host receptor for α-glucan has yet been identified, however, this polysaccharide is thought to play both direct and indirect roles in the immune response against *A. fumigatus*. Purified α-glucan inhibits both TLR2 and TLR4-mediated IL-6 production by PBMCs, although the molecular mechanisms underlying this observation remain unclear [136]. *A. fumigatus* mutants deficient in α-glucan produce conidia in which the normally-inert rodlet layer is covered by an amorphous layer of glycoproteins [137]. During germination, these conidia display increased amounts of surface exposed β-glucan and chitin [137], are more readily phagocytosed and killed by mouse alveolar macrophages, and induce higher levels of TNFα secretion by these cells in vitro [137]. Mutants lacking α-glucan are hypovirulent in mouse models of invasive aspergillosis, where conidia fail to germinate into hyphae [137], likely as a consequence of the dramatic alterations in cell wall structure. Collectively, these findings suggest that α-glucan plays an important role in masking cell wall PAMPs from immune recognition during early germination.

Although natural antibodies to α-glucan have not been described, a synthetic α-(1,3)-glucan pentasaccharide has been used successfully to generate anti-α-glucan antibodies [138]. These antibodies recognized native α-glucan on the surface of germinating *A. fumigatus* conidia, however, their potential as diagnostic tools and the ability of vaccination with this pentasaccharide or administration of anti-α-glucan antibodies to protect against *A. fumigatus* infection have yet to be evaluated.

### 3.4. Chitin

Chitin is located within the inner cell wall of *A. fumigatus* and plays an important role in structural integrity of the fungal cell. No host cell receptor for *A. fumigatus* chitin has been identified to date, however, the interaction of this glycan with a number of soluble factors has been implicated in the modulation of inflammation.

M-ficolin interacts with chitin on the surface of *A. fumigatus* young hyphae, and results in cleavage of the complement protein C4 by the protease MASP-2 [108]. Incubating A549 airway epithelial cells with M-ficolin and *A. fumigatus* extract resulted in elevated IL-8 production by these cells [108], suggesting that recognition of chitin by M-ficolin may alter inflammatory responses. Other members of the ficolin family H-ficolin (also known as ficolin-3), L-ficolin (also known as ficolin-2), and its murine ortholog A-ficolin have also been reported to recognize *A. fumigatus* conidia [139,140,141]. While the specific fungal ligands bound by these ficolins have not been defined, *N*-acetylglucosamine inhibits the binding of these soluble factors to *A. fumigatus*, suggesting that they may recognize chitin [139,140,141]. As with M-ficolin, treatment with H- and L-ficolin increased *A. fumigatus* conidia binding to A549 airway epithelial cells, and enhanced the release of IL-8 [141,142]. Intriguingly, both A- and L-ficolin treatment decreased the amount of IL-1β, IL-6, IL-8, and TNFα released by human monocyte-derived macrophages and neutrophils in response to conidia, despite increased levels of fungal uptake and killing [142]. H-ficolin enhanced activation of the lectin-dependent complement cascade in vitro, leading to increased deposition of C3 onto the conidial surface [141]. Studies using transgenic mice deficient in specific ficolins would greatly improve our understanding of the role of these proteins in the pathogenesis of *A. fumigatus* infection. 

Purified *A. fumigatus* chitin has been reported to induce anti-inflammatory effects via induction of IL-1Ra production by human PBMCs [143]. IL-1Ra production was mediated by anti-chitin IgG antibodies found in normal human serum interacting with FcγRII, leading to phagocytosis of chitin particles [143]. Interestingly, in the presence of TLR-2, -4, or NOD2 ligands, this response could be re-programmed to augment release of pro-inflammatory IL-1β [143]. Thus, the immune consequences of host-chitin interactions are likely context specific, and may vary during different stages of infection.

In animal models of fungal allergy, chitin exacerbates detrimental type-2 responses. Repeated exposure of mice to *A. fumigatus* conidia, or commercial crab shell chitin alone, was unable to induce a significant adaptive T_H_2 cell response, however, a combination of the two resulted in increased type-2 cytokines, such as IL-4, IL-5, and IL-13, eosinophilia, and high IgE antibody titers [63]. This phenomenon was initiated by C3 protein cleavage to generate C3a via the alternative pathway of the complement cascade, leading to a suppression of regulatory dendritic and T cells, and induction of allergy-promoting T_H_2 cells [63]. Similar findings were reported using purified crab shell chitin as an adjuvant when administering *A. fumigatus* culture filtrate intraperitoneally to mice [144]. Although priming with chitin prior to challenge reduced the release of IL-4, -5, and -13 in response to culture filtrate challenge, chitin priming still enhanced both eosinophil recruitment and the secretion of IgE antibodies [144]. Similarly, mice receiving repeated intranasal inoculation of conidia of an *A. fumigatus* strain with increased levels of exposed chitin resulted in enhanced eosinophil recruitment to the lungs, as compared to those exposed to the wild type strain Af293 [145]. The resulting T-helper cells were skewed to a type-2 phenotype, producing less IFN-γ, and more IL-4 [145]. This heightened type-2 response was detrimental to the host, as eosinophil-deficient, sensitized mice had significantly greater survival in a neutropenic model of *A. fumigatus* infection, as compared with wild type sensitized mice [145]. Taken together, these studies suggest that chitin elicits a predominantly type-2 immune response, although the host receptors involved in mediating this response remain undefined.

### 3.5. Galactosaminogalactan

Galactosaminogalactan (GAG) is a linear heteropolysaccharide composed of α-(1,4)-linked galactose and *N*-acetylgalactosamine (GalNAc) [146,147] that is found in the outer cell wall and extracellular matrix of hyphae [132]. Partial *N*-deacetylation of GalNAc residues renders the polymer cationic, and allows GAG to mediate adherence to the hyphal cell wall, as well as other anionic surfaces, such as human cells, plastic, and glass [101,148].

GAG has been described to play a number of passive and active roles in counteracting host immune responses. GAG conceals more immunoreactive cell wall components, such as β-glucan from host detection [101]. The cationic nature of GAG also protects the hyphae from neutrophil-mediated killing by repelling the cationic peptides found in neutrophil extracellular traps [149]. GAG also plays an active role in altering immune responses to *A. fumigatus*. A purified fraction of GAG was found to induce apoptosis of human neutrophils in whole blood samples [147], a process mediated by natural killer (NK) cells [150]. Soluble GAG induced neutrophil ROS production through an unknown mechanism, which in turn increased expression of MHC class I chain-related molecule A (MIC-A) on the surface of neutrophils [150]. MIC-A binding to NKG2D on the surface of the NK cells was then linked to Fas-dependent apoptosis via the caspase-8 pathway [150]. Purified GAG can also stimulate IL-1Ra secretion by human PBMCs, resulting in a suppression of T_H_1 and T_H_17 responses [151]. Finally, GAG has been reported to bind and activate human platelets, resulting in degranulation and exposure of CD62P on their surface [152]. The mechanism of these GAG-dependent direct effects on immune cells and the host receptors involved in GAG recognition are largely unknown.

As with other exopolysaccharides, GAG is antigenic in humans. Anti-GAG antibodies are present in up to 40% of human sera samples, even in the absence of prior history of *Aspergillus* disease [147]. Importantly, however, many of these antibodies also reacted with glycoproteins of *Campylobacter jejuni*, suggesting that the antibodies may have developed in response to other microbial glycans with structural similarity to GAG. This hypothesis, that GAG shares similarities with bacterial exopolysaccharides, is supported by a recent study demonstrating cross-species activity of bacterial glycoside hydrolases from *Pseudomonas aeruginosa* against *A. fumigatus* GAG [153].

### 3.6. Non-fumigatus Aspergillus Species

While *A. fumigatus* represents roughly 80% of all *Aspergillus*-related infections, it is not the most abundant species isolated from environmental sampling, suggesting that it expresses unique virulence factors to enable it to cause human infection. *Aspergillus nidulans*, while commonly isolated from the environment, is rarely associated with infections, except in patients with NADPH oxidase deficiency (chronic granulomatous disease, CGD). Although the reasons underlying this observation are not fully understood, it has been suggested that differences in cell wall GAG production may contribute to the pathogenicity of *A. nidulans* in patients with CGD. *A. nidulans* produces low levels of cell wall GAG due to reduced expression of the glucose 4-epimerase UgeB that synthesizes *N*-acetylgalactosamine, and as a result, is more sensitive to killing by neutrophil extracellular traps (NETs) [149]. Patients with CGD are unable to form NETs, and are thus lacking a key element of host defence against this pathogen. Low levels of cell wall associated GAG in *A. nidulans* have also been linked to increased production of pro-inflammatory cytokines by CGD PBMCs [154].

*Aspergillus terreus* is another uncommon cause of invasive aspergillosis [155]. Conidia of *A. terreus* display higher levels of β-glucan and galactomannan then do those of *A. fumigatus* [156]. Conidia of *A. terreus* were more rapidly phagocytosed by murine alveolar macrophages than those of *A. fumigatus*, and this was dependent on dectin-1 and mannose receptors [156]. Interestingly, unlike *A. fumigatus*, conidia of *A. terreus* failed to germinate within the phagolysosome, and persisted in a dormant but viable state, without inducing macrophage injury [156]. Another unique feature of *A. terreus* is its capacity to produce accessory conidia, in addition to those formed by phialides, both in vitro as well as in vivo. These accessory conidia are physically distinct, and exhibit higher levels of exposed β-glucan, resulting in increased detin-1 dependent production of pro-inflammatory cytokines by murine alveolar macrophages, in vitro and in vivo [157].

## 4. *Cryptococcus neoformans*

Infection with the yeast *Cryptococcus neoformans* is acquired by inhalation of dessicated yeast cells or basidiospores from fungi that are ubiquitous in the environment [158]. This exposure usually results in limited asymptomatic pulmonary infection; however, immunocompromised patients are at risk of developing pneumonia, disseminated disease, and meningitis. During infection, *C. neoformans* produces a large, mucoid capsule that surrounds and protects the yeast cells and is shed in large amounts during growth. The capsule is composed of three major components: glucuronoxylomannan, galactoxylomannan, and mannoproteins [159] that play key roles in the pathogenesis of cryptococcosis, by interfering with host recognition of β-glucans and mannoproteins within the cell wall [160], and cell phagocytosis, as well as by facilitating intracellular survival, replication, and extrusion through complex immunosuppressive and immunomodulatory mechanisms [161,162,163].

### 4.1. Glucuronoxylomannan

Glucuronoxylomannan (GXM) is the outermost and most abundant component of the capsule, forming >90% of its mass. GXM is composed of a poly-α-(1,3)-mannose backbone that can be 6-*O* acetylated, and substituted with β-(1,2)-linked glucuronic acid sidechains, and β-(1,2)- or β-(1,4)-linked xylose sidechains, depending on the serotype [158]. While the full repertoire of host receptors for this glycan remain poorly defined, several studies have suggested that GXM plays an important role in host–fungal interactions (reviewed in [164]). GXM is recognized by CD14, CD18, TLR2, and TLR4 in vitro; however, none of these pattern recognition receptors was absolutely required for serum clearance or hepatosplenic polysaccharide accumulation in vivo [165]. Knockout mouse models have shown a modest role for TLR2 or CD14, but not TLR4, on survival after cryptococcal infection [166]. Notably, deletion of the intracellular protein MyD88 had a much more significant effect on survival, fungal burden, and GXM levels in the lungs and sera after intranasal infection, suggesting that additional innate immune receptors that signal via this adaptor mediate the host response to GXM [166]. GXM can also directly interact with FcγRIIB, which has been implicated in *C. neoformans* uptake by phagocytic cells [167,168]; however, this interaction produces inhibitory signals that contribute to immune unresponsiveness [169].

Invasion and lysis of A549 airway epithelial cells by *C. neoformans* can be inhibited by anti-GXM antibodies [170]. Studies of leukocyte interactions with purified GXM in vitro have also reported a variety of host responses to this glycan. These include the production of TNFα, IL-6, IL-10, and RANTES by murine peritoneal macrophages [171], and induction of TGFβ, iNOS, and nitric oxide, leading to autophagy and ultimately apoptosis in RAW 264.7 macrophages [172]. Rat peritoneal macrophages also produce iNOS and undergo nitric oxide-dependent apoptosis in response to GXM [173]. Macrophage apoptosis was also dependent on CD18, FcγRII, and protein kinase C activation, but was associated with down-regulation of caspase-3 activity, suggesting that GXM-mediated apoptosis was mediated through a caspase-independent pathway [173]. GXM was reported to bind to CD18 on human neutrophils, raising the possibility that GXM may activate the β2-integrin apoptosis pathway, although apoptosis was not directly studied in this report [173].

In vitro studies also suggest that GXM can influence the adaptive immune response through inhibition of CD4 T cell activation [174]. Internalization of GXM by mouse BMDCs reduced their ability to induce antigen-specific T cell proliferation and IL-2 release [175]. Although this phenomenon was independent of cell death, a second study reported that GXM internalization by human monocyte-derived macrophages led to the Fas-mediated apoptosis of T cells [176]. GXM treatment also directly reduced T cell proliferation in response to PMA/ionomycin and anti-CD3 antibodies [177].

In addition to these direct effects on the immune response, GXM may also conceal ligands deeper in the capsule and cell wall from immune detection [160]. *C. neoformans* mutants lacking GXM induced higher levels of pro-inflammatory cytokines IL-12p40 and TNFα production by dendritic cells, than did wild type fungi [175].

*C. neoformans* strains deficient in GXM xylosylation are severely attenuated in virulence in a murine intravenous infection model [178], while strains deficient in GXM *O*-acetylation exhibit heightened virulence [179]; however, neither of these studies reported on the immune response mounted against these strains. Studies reporting the effects of purified GXM on immune responses in vivo have validated some of the in vitro observations discussed above. Intraperitoneal injection of purified GXM resulted in uptake by peritoneal macrophages and nitric oxide production in rats [173], as well as increased Fas/FasL-dependent peritoneal macrophage apoptosis in mice [172]. Intrapulmonary administration of GXM led to upregulation of pulmonary iNOS in rats [173], and induced IL-10 and TNFα secretion in mice [171]. Co-administration of GXM with chitin elicited higher levels of IL-10, IL-17, and TNFα release, than either glycan alone [180]. Thus, it is possible that this synergistic response to multiple glycans enhances the specificity of the immune response to fungal pathogens, while avoiding deleterious responses to environmental glycans.

### 4.2. Galactoxylomannan

A second important polysaccharide found within the *C. neoformans* capsule is galactoxylomannan (GalXM), composed of an α-(1,6)-galactose backbone with trisaccharide branches of mannose-α-(1,3)-mannose-α-(1,4)-galactose-β-(1,3) [158]. These branches may be further xylosylated through β-(1,2) and β-(1,3) linkages [158]. GalXM is located deep within the capsule, adjacent to the cell wall, and forms 5–10% of its mass [159].

As with GXM, the effects of purified GalXM on leukocyte responses have also been studied. GalXM binds CD18 on human neutrophils [181], and induces TGFβ, TNFα and iNOS production by RAW macrophages, leading to autophagy and apoptosis mediated by Fas/FasL interactions [172]. GalXM has a more marked effect on the cells of the adaptive immune system, as compared to GXM. Purified GalXM induces the release of IFN-γ and IL-10 [182] by human PBMCs, suppresses purified human T lymphocyte proliferation, and directly induces Fas/FasL-dependent T cell apoptosis. GalXM-induced apoptosis of human T cells is dependent on interactions with CD7 and CD43 (also known as leukosialin or sialophorin) that activate both extrinsic and intrinsic apoptosis pathways through caspase-8 cleavage [183,184]. As with GXM, peritoneal injection of GalXM also increases Fas/FasL-dependent apoptosis of resident macrophages [172]. Administration of GalXM, in this model, was associated with reduced inflammatory cytokine expression by splenocytes, and caspase- and Fas-dependent apoptosis of antigen-specific B cells, resulting in a state of immune paralysis [185]. While virulence studies using GalXM-deficient *C. neoformans* have revealed these strains to be hypovirulent in vivo [186], detailed studies of the GalXM-specific immune response have yet to be performed.

### 4.3. Mannoproteins

Mannoproteins comprise a small fraction (<1%) of the *C. neoformans* capsule. Mannosylation of these proteins is extensive, such that mannose residues comprise 80–90% of the weight of these molecules [187], and is required for mannoprotein-mediated T cell stimulation [188]. Like GalXM, mannoproteins are situated deep within the capsule, which conceals them from immune recognition [159,189].

Unlike GalXM and GXM that have complex immunosuppressive and immunomodulatory effects, mannoproteins appear to induce a predominately pro-inflammatory immune response [190]. In studies using purified capsule components, mannoproteins were found to be the strongest inducers of human PBMC proliferation [191] and IL-6 production [192]. Proliferation of PBMCs in response to mannoproteins could be inhibited by antibodies against ICAM-1, LFA-1, and MHC class II, suggesting that this response is dependent on antigen recognition [191]. Human dendritic cells have been found to internalize and process mannoproteins via the mannose receptor, leading to the maturation and activation of these cells [193]. Mannoprotein-activated dendritic cells produced IL-12 and TNFα, and were efficient at stimulating CD8 and CD4 T cell proliferation, and T cell differentiation towards a T_H_1 phenotype [194]. Mice lacking the mannose receptor died significantly faster than wild type mice, with higher lung fungal burdens at 4 weeks after infection, and they displayed impaired CD4^+^ T cell responses to mannoprotein [195]. Mannoprotein-dependent T_H_1 responses also provided cross-fungal immunity against lethal *C. albicans* challenge [196]. Thus, although mannoproteins are highly effective at inducing protective antifungal immune responses, their abundance and location deep within the capsule likely limits their role as activators of protective antifungal immune responses.

### 4.4. Cryptococcus gattii

While *C. neoformans* typically only causes disease in immunosuppressed individuals, such as AIDS patients, a recently emerged strain of *Cryptococcus gattii* isolated from the Pacific Northwest has been reported to infect and cause disease in immunocompetent hosts [197]. Studies of the differences in virulence, that could contribute to this difference in host requirement, found that this strain of *C. gattii* induced a much lower inflammatory response, as compared with *C. neoformans* [198,199,200]. Immunocompetent mice infected with *C. gattii* exhibited limited cellular recruitment to the site of infection, and depletion of CD4-positive cells had no effect on survival, while mice infected with *C. neoformans* displayed a robust cellular immune response, and CD4 cell depletion significantly reduced survival time [200]. Coincubation of the two *Cryptococcus* strains with dendritic cells, in vitro, found that while *C. neoformans* stimulated a strong IL-6 response, *C. gattii* failed to induce IL-6 production by these cells [200]. This difference in cytokine response was related to partial deacetylation of *C. gattii* GXM, that was absent in *C. neoformans*. Chemical deacetylation of GXM from both species abolished their recognition by dendritic cells [200]. The mechanism underlying this deacetylation-dependent difference in host response is unknown, though it is interesting to hypothesize that there are similarities between this process and the role of partially deacetylated *A. fumigatus* GAG in immune evasion. Complement protein C3 also binds more strongly to GXM of *C. neoformans* than *C. gattii*, although this difference in C3 binding was attributed to differences in polysaccharide branching, rather than deacetylation [201].

## 5. *Histoplasma capsulatum*

*Histoplasma capsulatum* is a thermally dimorphic fungus that is a primary human pathogen [202]. *H. capsulatum* grows in a filamentous form in the environment, where it produces conidia that can be disseminated, particularly during excavation or other physical disruption [203]. Following inhalation, and exposure to higher body temperatures, conidia develop into their yeast form, causing pulmonary and disseminated infection [202]. Studies of immune interactions with *H. capsulatum* have been largely limited to elucidating the strategies by which this organism conceals β-glucans.

### Alpha-(1,3)-Glucan

As with *A. fumigatus*, α-(1,3)-glucan is found in the outer cell wall of *H. capsulatum*, where it can mask β-glucans from detection by dectin-1. Strains deficient in α-glucan were significantly attenuated in their ability to kill murine P388D1 macrophage-like cells in co-culture [204], and were rapidly phagocytosed by these cells, leading to increased dectin-1-dependent TNFα secretion [205]. Interestingly, α-glucan-deficient strains of *H. capsulatum* and wild type *C. albicans* induced similar levels of TNFα production [205], suggesting that the efficiency of β-glucan masking by *H. capsulatum* may contribute to the success of this organisms as a primary pathogen. Masking of β-glucans by α-glucan is not universal among strains of *H. capsulatum*, as α-glucan-deficient strains (chemotype I), have been reported [206]. Although these yeast bind dectin-1 during log phase growth, dectin-1 binding is lost during stationary phase [206]. While the effects of deleting α-glucan synthase in chemotype I strains on stationary phase masking of β-glucans has not been studied, inhibition of α-glucan synthase function via RNA interference resulted in no decrease in virulence, either in vitro or in vivo [206]. These findings suggest that β-glucan-masking in chemotype I strains is α-glucan independent [206,207]. Despite the absence of α-glucan, chemotype I yeast retain their ability to be internalized by, and kill, mouse macrophages [206], although less rapidly than α-glucan-sufficient strains [208].

In addition to α-glucan masking of β-glucans from immune recognition, *H. capsulatum* also secretes β-glucanase enzymes to further limit surface exposure of this glycan [207,209]. Strains from both chemotypes that are deficient in β-glucanase exhibit increased recognition by dectin-1, resulting in greater amounts of TNFα and IL-6 release by murine peritoneal macrophages during infection [207,209]. In α-glucan sufficient strains, deletion of α-glucan synthase resulted in a greater increase in dectin-1 binding than did deletion of β-glucanase, although the effects were additive [207]. These observations suggest that while the masking of β-glucan with the α-glucan layer is the dominant mechanism of immune evasion, the two strategies are complementary. It has been suggested that the role of the secreted β-glucanase is to “trim” off any exterior β-glucan that remains exposed beyond the α-glucan coat surrounding the yeast cell [207].

Studies in mouse models have mirrored these in vitro findings. Chemotype II *H. capsulatum* strains deficient in α-glucan were significantly attenuated in virulence and exhibited reduced ability to disseminate beyond the lung in a mouse model of pulmonary infection [206]. In contrast, naturally α-glucan-deficient chemotype I strains remained virulent in mouse models of pulmonary histoplasmosis, and deletion of α-glucan synthase in these strains had no effects on overall virulence [206]. Although both chemotypes of *H. capsulatum* are capable of causing lethal diseases, studies using a sub-lethal pulmonary infection mouse model revealed differences in the immune response to these two strain types. Infection with α-glucan-deficient chemotype I *H. capsulatum* was associated with greater pulmonary levels of IFN-γ, IL-1β, IL-12, and TNFα, in association with increased weight loss, more severe pathology in lung histology, and higher pulmonary fungal burden later in the infection [208]. These differences in the kinetics of infection further support the hypothesis that chemotype I strains utilize unique virulence factors to support infection. Studies in mice have also confirmed the importance of the β-glucanase in virulence [207]. Mice infected intranasally with β-glucanase-deficient *H. capsulatum* strains had significantly lower pulmonary fungal burden than mice infected with the wild type parent strain, regardless of chemotype [207]. This difference in fungal burden was not observed in dectin-1-deficient mice, confirming that these alterations in virulence were due to differences in β-glucan recognition [207].

## 6. Thoughts and Perspectives

Significant progress has been made in recent years regarding the study of fungal exopolysaccharides and the effects they have on the host immune system. These findings have led to a range of new therapeutic strategies targeting fungal polysaccharides [210]. In addition to the currently licensed echinocandins, inhibitors of glycosyl phosphatidylinositol synthesis that prevent incorporation of mannoproteins into the fungal cell wall, are currently in clinical trials [211]. Other efforts include generating antibodies against *C. albicans* mannans [212], cell wall glycoproteins [213], and *A. fumigatus* α-glucan [138], to test the vaccine potential of these cell wall components. Finally, we have recently reported the use of microbial glycoside hydrolases to degrade *Aspergillus* GAG, increase β-glucan exposure, and reduce virulence in a mouse model of invasive aspergillosis [153,214]. While many of these approaches remain in the early experimental phase, the therapeutic potential of the fungal cell wall is enormous.

Another promising avenue of research is to exploit immunomodulatory properties of fungal exopolysaccharides as treatments for inflammatory and autoimmune diseases. For example, *A. fumigatus* GAG has been proposed as a treatment for colitis through the induction of IL-1Ra [151]. Similarly, *C. neoformans* GalXM has been investigated as a treatment for rheumatoid arthritis, due to its ability to induce T cell apoptosis and inhibit IL-17 production [215].

Despite the tremendous advances in our understanding of the host immune response to fungal polysaccharides, significant challenges remain. Purification and characterization of cell wall polysaccharides remains in its infancy, and it is highly likely that variations in polymer length and post-synthetic modifications have a major impact on the host recognition and response to these molecules. Further, as evidenced by the results of studies using chitin and GXM [180], the immune response to combinations of polysaccharides may differ from those observed with isolated single polysaccharides. The study of fungal mutants that are deficient in specific polysaccharides is helpful, however, compensatory changes in cell wall composition through activation of the cell wall integrity and other pathways can lead to misleading results. Further, strain-, species-, and growth condition-dependent differences in cell wall composition may limit the generalizability of observations from in vitro and in vivo studies. Lastly, our ability to study the dynamics of cell wall polysaccharide synthesis and modification during infection remains in its infancy. A combination of experimental approaches, and the development of new tools to assay, manipulate, and quantify polysaccharide production in vitro and in vivo are required to move the field forward and maximize the therapeutic potential of these microbial molecules.
